# Effects of Essential Oils and Hydrolates on the Infectivity of Murine Norovirus

**DOI:** 10.3390/v15030682

**Published:** 2023-03-04

**Authors:** Loredana Cozzi, Teresa Vicenza, Roberta Battistini, Chiara Masotti, Elisabetta Suffredini, Simona Di Pasquale, Marie-Laure Fauconnier, Carlo Ercolini, Laura Serracca

**Affiliations:** 1 Department of Food Safety, Nutrition and Veterinary Public Health, Istituto Superiore di Sanità, 00161 Rome, Italy; 2 Department of La Spezia, Istituto Zooprofilattico Sperimentale del Piemonte, Liguria e Valle d’Aosta, Via degli Stagnoni 96, 19100 La Spezia, Italy; 3 Laboratory of Chemistry of Natural Molecules, Gembloux Agro-Bio Tech, University of Liège, Passage des Déportés 2, 5030 Gembloux, Belgium

**Keywords:** *Citrus limon*, *Thymus serpyllum*, *Thymus vulgaris*, natural compounds, virucidal activity, norovirus

## Abstract

The use of natural substances with antiviral properties might reduce foodborne viral diseases. In this study, we evaluated the virucidal effect of *Citrus limon* and *Thymus serpyllum* essential oils (EOs) and of *Citrus Limon*, *Thymus serpyllum* and *Thymus vulgaris* hydrolates on murine norovirus (MNV), a human norovirus surrogate. To assess the virucidal effect of these natural substances, the reduction in viral infectivity was estimated by comparing the TCID_50_/mL of untreated viral suspension and the viral suspension treated with hydrolates and EOs at different concentrations. The results showed a natural loss of infectivity of the untreated virus after 24 h of approx. 1 log. The EO (1%) of *T. serpyllum*, and hydrolates (1% and 2%) of *T. serpyllum* and *T. vulgaris* immediately caused a reduction in MNV infectivity of about 2 log but did not provide a further significant decrease after 24 h. Instead, the EO (1%) and hydrolate (1% and 2%) of *C. limon* exerted an immediate reduction in the viral infectivity of about 1.3 log and 1 log, respectively, followed by a further reduction in infectivity of 1 log after 24 h for the hydrolate. These results will allow for the implementation of a depuration treatment based on the use of these natural compounds.

## 1. Introduction

Essential oils (EOs) and hydrolates are natural products of plants that can be extracted from the leaves, petals, stems, seeds or roots of plants through hydro-distillation or steam distillation [1]. EOs are the principal products obtained during this process; they are a complex mixture of many different lipophilic, low molecular, aromatic, highly volatile substances which belong to several different chemical classes, including alcohols, ethers, aldehydes, ketones, esters, amines, amides, phenols, heterocycles, and terpenes [1,2]. Hydrolates are a mixture containing a variable quantity of EO (usually less than 1 g/L) and volatile, water-soluble, secondary metabolites [3]. The aromatic profile of hydrosols can significantly differ from the respective EOs because of their lack of hydrophobic, water-insoluble isoprenoid compounds (hydrocarbons) [4]. In the past, hydrosols have been defined as waste products of steam distillation. Recently, many researchers have reconsidered them, analyzing their antimicrobial antifungal and antioxidant capacity [5]. EOs are widely reported as possessing antimicrobial, antimycotic, antiparasitic and insecticidal properties against human pathogens [6,7,8,9,10,11]. The antiviral efficacy of EOs has also been evaluated [12]. In recent years, EOs have more often been investigated against enveloped viruses [13,14,15], while to date, only limited research has looked at the efficacy of EOs against non-enveloped viruses [16,17,18]. While the scientific literature on essential oils is relatively abundant, much less is known about hydrosols [3] and to date, the antiviral effect of hydrosols against human enteric viruses has scarcely been studied [19]. Enteric viruses have been recognized as an important cause of foodborne disease in developed countries. Noroviruses (NoV) have been one of the major responsible agents of viral gastroenteritis worldwide and the main cause of foodborne illness in Europe and US associated with the consumption of oysters [20]. Shellfish depuration as currently applied is not effective for reducing NoV. It is therefore important to identify different post-harvest intervention strategies to reduce this pathogen in oysters and to increase the safety of this food.

NoV is a small (30–35 nm) non-segmented and non-enveloped RNA virus belonging to the *Caliciviridae* family [20]. NoV does not multiply in vitro in cell cultures; consequently, in laboratory tests, the evaluation of the inactivation efficiency of NoV through any substance or treatments continues to rely largely on the use of easily cultured surrogates with relatively close structural and genetic similarities to NoV, such as feline calicivirus (FCV), murine norovirus (MNV) and Tulane virus (TV) [21,22,23,24]. MNV is considered as the best surrogate for human NoV as it is transmitted via the fecal-oral route, and it can mimic the survival and inactivation of NoV [25]. EOs and hydrolates might offer the possibility of preventing and controlling foodborne diseases and it is worth evaluating their use as a possible additive during the purification treatment of shellfish. 

*Thymus* and *Citrus* species plants constitute two of the main sources of essential oil, which are extensively studied for their potential uses in the food industry [26,27]. *Thymus vulgaris* L. and *Thymus serpyllum* L. or wild thyme of the family *Lamiaceae* are aromatic flowering plants originating from the Mediterranean region which contain high amounts of EOs rich in polyphenolic compounds—phenolic acids or flavonoids [28,29]. *T. serpyllum* and *T. vulgaris* extracts possess antibacterial, antimicrobial, antifungal, and insecticidal effects [30,31,32,33]. *T. serpyllum* EO also has an inhibitory effect against biofilm-forming microorganisms, but less is known regarding its antiviral properties [34,35]. Regarding hydrosols, only the antiviral activity of *Thymus vulgaris* against porcine reproductive and respiratory syndrome virus (PRRS) was indicated [36]. *Citrus limon* (L.) Osbeck (Lemon) is among the most important species of genus *Citrus* belonging to the *Rutaceae* family, which includes about 140 genera and 1300 species [37]. Essential oils were composed of 85–99% of volatile components, including monoterpene (limonene), sesquiterpenes, and hydrocarbons; their oxygenated products include aldehydes (citral), ketones, acids, alcohols (linalool), and esters [38]. Limonene (1-methyl-4-(1-methylethenyl)), which is the main ingredient of lemon essential oil, is one of the most common terpenes in nature and is widely found in the volatile oils of various plants [39]. Limonene has broad application prospects in antibacterial and food preservation due to its broad-spectrum bactericidal activity, safety, and low toxicity [40]. Limonene d-limonene and lemon essential oil were found to have antimicrobial activities against foodborne bacterial and fungal pathogens [41,42,43,44]. However, few studies have investigated the antiviral properties of limonene and lemon essential oil [14,18,45,46].

In this study we investigated in vitro the virucidal efficacy of *Thymus serpyllum*, *Thymus vulgaris* and *Citrus limon* hydrolates and *Thymus serpyllum* and *Citrus limon* EOs against murine norovirus. The results obtained will be used to implement a depuration treatment of shellfish based on the use of these natural compounds.

## 2. Materials and Methods

### 2.1. Virus Strain and Cell Line

The MNV-1 strain was replicated in RAW 264.7 cells, cultured in Dulbecco’s Modified Eagle’s Medium (DMEM) supplemented with 1% glutamine, 1% non-essential amino acids and 2% fetal bovine serum (FBS) incubated in 5% CO_2_ at 37 °C. EuroClone (Milan, Italy) provided all cell culture media. The viral suspension was prepared via freeze and thaw lysis (1 cycle) of infected monolayers, clarified using low speed centrifugation (800× *g*) to remove residual debris, and then divided in aliquots and stored at −80 °C until use. The obtained viral stock suspension had a final concentration of 5.4 ± 0.1 log TCID_50_/mL, calculated by determining the 50% tissue culture infectious dose using the Reed and Muench method [47] using tenfold serial dilutions in 24-well plates.

### 2.2. Hydrolates and Essential Oils (EOs)

Commercially available essential oils from *Citrus limon*, *Thymus serpyllum* (Flora Srl, Pisa, Italy) and hydrolates from *Citrus limon*, *Thymus serpyllum* and *Thymus vulgaris* (I Segreti delle Erbe, Netro, Bl, Italy) were used in this study.

The composition of the EOs were analyzed through gas chromatography/mass spectrometry (GC-MS) using an Agilent GC system 7890B (Agilent, Santa Clara, CA, USA) fitted with a split-splitless injector and coupled to an Agilent MSD 5977B detector. The EOs were diluted in analysis grade hexane (10 µL /10 mL) and one microliter of EOs solution was injected. The analytical conditions were fixed as follows: injection mode: splitless at 280 °C; HP-5MS capillary column (Agilent, Santa Clara, CA, USA) (30 m × 0.25 mm, df = 0.25 µm); temperature program: from 40 °C (2 min) to 300 °C (5 min) at a rate of 6 °C/min. The carrier gas was helium at a flow rate of 1.2 mL/min. The mass spectra were recorded in electron ionization mode at 70 eV (scanned mass range: 35–400 *m*/*z*). The source and quadrupole temperatures were fixed at 230 °C and 150 °C, respectively. The identification was performed on the basis of chromatographic retention indices (RI) and through comparison of the recorded spectra with a computed data library (Pal 600K^®^). Experimental retention index (RI) of the compounds were calculated following the injection of a mixture of n-alkanes C8-C20 (Sigma Aldrich, Darmstadt, Germany). Results were reported as a percentage of the total chromatographic area.

Hydrolates were analyzed via SPME-GC-MS using a 50/30 µm DVB/CAR/PDMS (Supelco, Bellefonte, PA, USA) fiber that was preconditioned according to the instructions of the manufacturer. Extraction was performed for 1 min at 30 °C and the injection was realized at 280 °C, while the chromatographic conditions were the same as for essential oils.

### 2.3. Cytotoxicity Determination of Hydrolates and EOs on Cells

Preliminary tests were performed on cell cultures to identify the EO and hydrolate concentrations that did not produce a cytotoxic effect. Hydrolate solutions were prepared in serum-free DMEM, while EOs, due to their hydrophobicity, were emulsified in 33% sunflower oil, 0.1% tween 80 physiological saline solution. Solutions of the natural compounds at different concentrations (0.0125%, 0.025%, 0.05%, 0.25%, 0.5%, 1%, 2% *v*/*v*) were treated overnight at 4 °C with antibiotic/antimycotic solution (Euroclone) and 1 mL of each concentration was assayed on 24–48 h cell monolayers in a 25 cm^2^ flask. The monolayers were incubated for 1 h in 5% CO_2_ at 37 °C. Thereafter, cells were washed twice with Dulbecco’s phosphate buffer solution (DPBS, EuroClone) and maintained with DMEM supplemented with 2% of FBS for 4 days in 5% CO_2_ at 37 °C. A cytotoxicity effect was qualitatively determined via visual inspection under optical invertoscope. No morphological changes of monolayer, such as lyses, granulation, condensation, vacuolization in the cytoplasm, darkening of cell boundaries and cell detachment, were to be shown.

### 2.4. Virucidal Effect of Hydrolates and EOs

The highest EO concentration (1% *v*/*v*) and hydrolate concentrations (2% *v*/*v*) that did not exert a cytotoxic effect were used to treat the MNV-1 suspension to evaluate the virucidal effect. Furthermore, to assess if lower concentrations allowed comparable or proportionate reductions in viral infectivity, EOs and hydrolates were also tested using 0.5% and 1% *v*/*v* concentrations, respectively. In detail, virus aliquots (titre of 5.4 ± 0.1 log TCID_50_/mL) were treated and analyzed immediately (t = 0) and after 24 h (t = 24) of incubation at 20 ± 2 °C [48] with each of the EOs or hydrolates. Untreated viral suspensions, incubated for the same time and at the same temperature, were used as positive controls, while hydrolates solutions and EOs emulsions were used as negative controls. Each treatment condition was assayed in triplicate. Viral titrations were performed by determining the TCID_50_/mL. Briefly, 100 µL of serial tenfold dilutions of each sample was assayed in 24-well tissue culture plates containing 24–48 h monolayers of RAW cells, and incubated for 1 h in 5% CO_2_ at 37 °C. After that, the wells were washed twice with 200 µL of PBS and 500 µL of DMEM, supplemented with 2% of FBS, were added to each well; incubation was carried out for up to 6 days in 5% CO_2_ at 37 °C with daily visual inspection. The reduction in viral infectivity was estimated as log reduction value (LRV) by calculating the log_10_ N_0_/ N_1_, where N_0_ is the titre for untreated viral suspension and N_1_ is the titre for treated viral suspension.

### 2.5. Statistical Analysis

For each treatment, the average and standard deviation of the triplicate analysis were calculated. The statistical significance of differences between treated and untreated samples was determined through one-way analysis of variance (ANOVA) with Bonferroni post hoc comparisons, with a significance level of *p* < 0.05 (GraphPad Prism v9.5.0, software San Diego, CA, USA).

## 3. Results

### 3.1. Chemical Composition of the Hydrolates and EOs 

Table 1, Table 2 and Table 3 reports the percentage of each component of the EOs and hydrolates, identified using GC/MS and SPME-GC-MS analysis. In *T. serpyllum* EO, a total of 31 compounds were identified, while 10 were found in *T. serpyllum* hydrolate. The two main compounds identified were carvacrol and linalool with respective percentages of 53.96% and 11.88% in EO and 58.67% and 17.11% in the hydrolate. In EO, thymol (5.74%) and terpinene (4.42%) were also abundant compounds, while cymene (11.23%) and terpinene (6.04%) were abundant in the hydrolate (Table 1). In *T. vulgaris* hydrolate, 12 compounds were identified, with thymol (84.01%) and carvacrol (7.55%) being the most abundant ones. In *Citrus limon*, the EO and the hydrolate were, respectively, composed of 25 and 12 identified compounds, with the main compounds being very similar: limonene (53.37–53.45%), beta-pinene (18.09–20.60%) and gamma-terpinene (12.55–14.03%) (Table 3). 

### 3.2. Virucidal Effects of Hydrolates and EOs

The results of the inactivation assays are summarized in Table 4. The untreated MNV-1 viral stock (titre of 5.4 ± 0.1 log TCID_50_/mL) displayed a natural decay of infectivity during the 24 h incubation at 20 ± 2 °C, with a reduction in the infectious titre of 1.2 log TCID_50_/mL. The MNV-1 aliquot treated with 1% *Citrus limon* hydrolate solution showed an immediate reduction (t = 0) in MNV-1 infectivity of 0.9 log TCID_50_/mL; the same reduction of 0.9 log TCID_50_/ml was observed after 24 h. A similar decrease was also obtained with 2% *Citrus limon* hydrolate (1.1 log TCID_50_/mL inactivation immediately and a reduction of 1.1 log after 24 h). Conversely, the treatments with *Thymus vulgaris* and *Thymus serpyllum* hydrolates provided the highest instantaneous reduction in viral infectivity: 1.9 and 2.0 log TCID_50_/mL reduction was achieved with 1% and 2% *Thymus vulgaris* hydrolate, respectively, while the corresponding *Thymus serpyllum* concentrations obtained a 2.0 and 1.8 log TCID_50_/mL reduction in infectious MNV-1. After 24 h of treatment, the obtained viral titres of MNV-1 were almost identical. A comparable behavior was observed in the treatment with EOs. The virus aliquots treated with 1% *C. limon* showed an immediate reduction (t = 0) of MNV-1 infectivity of about 1.3 log TCID_50_/mL and a reduction of 0.8 log after 24 h. In contrast, 1% *Thymus serpyllum* induced a loss of infectivity of 1.9 log TCID_50_/mL immediately, and the viral titre was unaffected after 24 h of treatment. A lower concentration of both EOs (0.5% *v*/*v*) showed no effect either immediately or after 24 h of treatment. 

## 4. Discussion

In this study, we evaluated the in vitro virucidal activity of *C. limon, T. serpyllum* and *T. vulgaris* hydrolates and of *C. limon* and *T. serpyllum* EOs on non-enveloped human NoV surrogate, MNV-1, to assess their potential use as a depuration treatment in the shellfish industry for the reduction in NoV exposure risks in oysters. For this purpose, we have decided to keep the virus in contact with the EOs and hydrolates for up to 24 h, because this is the time routinely used for the purification of shellfish. 

The results obtained clearly showed greater and faster virucidal activity exerted by *T. vulgaris* and *T. serpyllum* compared to *C. limon.* In fact, an immediate reduction of about 2 log was observed after treatment with hydrolates of *T. vulgaris* and *T. serpyllum* both 1% and 2% and with *T. serpyllum* EO at 1%. Instead, the reduction exerted by *C. limon* hydrolate (both 1% and 2%) and *C. limon* EO (1%) was of about 1 log both immediately and after 24 h of treatment. Considering the natural decay of MNV-1 infectivity after 24 h at 20 °C, *C. limon* probably has a lower virucidal efficacy than thyme. In any case, the reduction in the viral infectivity for all the compounds was >90% at t = 0, in detail it was about 99% for the thyme and 92% for the lemon. Moreover, it is important to underline that most of the virucidal effect of these natural compounds is exerted immediately after contact with the virus; thus, longer times do not involve further significant reductions in viral infectivity.

These results refer to the virucidal activity on MNV-1 of the natural compounds tested in this study in the adopted experimental conditions (24 h at 20 °C), considering the possibility of using them in a shellfish purification system. Other authors have conducted similar studies on MNV-1 using essential oils from other plants [49,50]. These authors have shown that the antiviral activity of the EOs is closely linked to the experimental conditions used and to the type of virus.

The antiviral activity of essential oils and hydrolates may be related to the presence of bioactive compounds. *T. serpyllum* and *T*. *vulgaris* contain significant amounts of monoterpenes, such as thymol and carvacrol, while limonene is the ingredient found most commonly in *C. limon*. From the analysis of the chemical profile of the two hydrolates of thyme used in our study, it is interesting to note that despite the hydrolates having a different concentration of thymol and carvacrol, they presented comparable results for the inactivation of MNV. *T. vulgaris* hydrosol contains 84% thymol and 7.5% carvacrol, while *T. serpyllum* hydrosol contains 58% carvacrol and does not contain thymol (Table 1 and Table 2). The chemical composition of hydrosols and EOs varies according to many factors, including seasonal variations, plant maturity and genetics [2]. The chemical diversity of the genus *Thymus* EO has been reported in several studies showing the existence of different chemotypes on the basis of major oil components [30,51,52]. Thymol and carvacrol are two of the most common chemotypes of the *Thymus* genus. These compounds are generally considered significant antimicrobial agents due to their richness in phenolic compounds [53]. They showed strong antimicrobial activity against a wide range of microorganisms [54,55,56,57], with a strong synergistic effect when applied together [58]. Carvacrol has been shown to inhibit viruses responsible for food-borne diseases, such as the human rotavirus, or non-enveloped murine norovirus (used as a surrogate of the human norovirus) as well as others, i.e., the human respiratory syncytial virus and acyclovir-resistant herpes simplex virus type 1 [17,59]. However, thymol and carvacrol protect against HIV-target cell fusion [60] but do not show antiviral activity against coxsackievirus B3 [61]; additionally, carvacrol showed low antiviral properties against Phi6 virus, which has been considered a suitable bacteriophage surrogate for coronaviruses.

Gilling et al. [17] determined the antiviral efficacy of carvacrol. Carvacrol was tested at concentrations of 0.25% and 0.5%. Both concentrations resulted in a statistically significant reduction in MNV within 15 min in comparison with the control sample.

Thymol was also effective in reducing the titer of norovirus surrogates in a dose-dependent manner. Thymol at concentrations of 1 and 2% reduced MNV titers by 1.66 and 2.45 log TCID_50_/mL, respectively [50].

The phytochemical composition of *T. serpyllum* EO and hydrolate used in this study also showed a high concentration of linalool (11.88% and 17.11%) and cymene (8.3% and 11.23%). Linalool (2,6-dimethyl-2,7-octadien-6-ol) is an aromatic monoterpene alcohol that is widely found in thyme [62]. Several studies have shown an important anticarcinogenic, anti-inflammatory and antibacterial activity [63,64]; however, there are no studies on its possible antiviral activity.

Cymene is considered the most important monoterpene compound occurring in aromatic plants, such as thyme and oregano. This compound shows a variety of biological activities which include antioxidant, antinociceptive, anti-inflammatory, anxiolytic, anticancer and antimicrobial activities [65]. To date, few studies have investigated the antiviral activity of cymene and none against MNV [66,67,68]. 

Both the EO and the hydrolate of *C. limon* used in this study contained a high concentration of limonene of about 53%. Limonene is the ingredient found most commonly in both lemon essential oil and hydrosol, and it has antimicrobial and antifungal activity against many foodborne pathogens. However, to date, there are still few studies investigating the antiviral properties of limonene, none of which involve enteric viruses [14,45,46]. In one study, the effect of *C. limon* at 0.5% against hepatitis A virus (HAV) infectivity showed a statistically significant reduction of 2.84 log TCID_50_/mL in HAV titer [18]. This is the first study to evaluate the effectiveness of hydrosols of *T. serpyllum, T. vulgaris* and *C. limon* against MNV infectivity. To date, only *T. vulgaris* hydrosol has been evaluated on porcine reproductive and respiratory syndrome virus (PRRS) [36], where the results showed a significative reduction in PRRSV load in vitro (*p* < 0.05).

There are not many studies investigating how EOs and their active compounds act on viruses; some show they have an action on the viral capsid, but it is difficult to determine whether the reductions in virus infectivity are due to actual damage to the viral particles or to a simple inhibition of virus uptake in host cells. For example, in many cases, viral RNA was not damaged although the virus was no longer infectious [69].

Plant metabolites may exhibit various mechanisms of antiviral activity; they can cause a direct virucidal effect against non-enveloped virus ssRNA by degrading the capsid or viral nucleic acid. Plant-derived compounds can also bind to the surface of the virus without destroying the proteins in the capsid, thus, interfering with its adsorption to host cells [70,71].

In non-enveloped viruses, the capsid protects the integrity of the viral nucleic acid. Viral RNA may remain intact, while changes in the structure of the capsid may deactivate the virus [72,73]. Modification of the virus capsid is one of the mechanisms that can lead to the inhibition of the virus adsorption process, which is associated with its deactivation. In the case of MNV, the results obtained by Gilling et al. [17] indicate that oregano oil containing a high concentration of carvacrol and carvacrol itself affect the complete loss of the integrity of the capsid [17].

Therefore, further studies are needed to understand the molecular mechanism of action of these natural compounds.

Our results show that the EOs and hydrolates of lemon and thyme were able to significantly decrease MNV infectivity during the in vitro experiments within 24 h. Therefore, the use of these substances in the shellfish purification to reduce the risk of exposure to NoV looks promising and worth investigating. Between the EOs and hydrolates, the latter are the most suitable for this purpose as they are easily miscible in water. In vivo application, however, may have many problems to solve, such as the toxicity of hydrosols to oysters and the ability to reduce viral infectivity even when the virus is inside the oyster. It must also be considered that hydrosols have an intense taste and smell, which could modify the taste and aroma of the oysters. Studies will therefore also be needed to evaluate the organoleptic impact of these treatments on mollusks.

## 5. Conclusions

Among the natural compounds, EOs obtained from plants have more often been investigated for their antimicrobial and antifungal activity. In recent years, antiviral activity has also been studied both with regard to EOs and hydrolates. Even if the hydrolates are the secondary products of the distillation process for the EOs extraction from plants, they maintain those compounds that have antiviral activity. Considering that hydrolates are in an aqueous solution, their use can be advantageous swhere essential oils cannot be used due to their hydrophobicity. The results of this study improve the knowledge about the antiviral activity of EOs and hydrolates and their potential use in food sanitation.

## Figures and Tables

**Table 1:** Phytochemical composition (%) of essential oil (EO) and hydrolate (H) of *Thymus serpyllum* (Ts) used in this study, via GC-MS. RI exp: retention index calculated with our experimental results; RI ref: retention index found in the literature for the same compound (source: Pherobase).

No. Peak	Compound Name	RI Exp.	RI Ref.	EO (Ts)	H (Ts)
1	Butanoic acid. 2-methyl-. methl ester			0.16	-
2	Beta-thujene	930	931	0.42	-
3	Alpha-pinene	937	939	1.02	1.25
4	Camphene	952	953	0.24	0.31
5	Beta-pinene	979	975	0.18	0.26
6	1-octen-3-ol	982	979	0.32	-
7	Beta-myrcene	992	991	1.59	2.02
8	3-octanol	996	993	0.16	-
9	Alpha-phellandrene	1005	1005	0.28	0.39
10	3-carene	1011	1011	0.12	-
11	4-carene	1019	1013	1.86	-
12	Cymene	1028	1026	8.3	11.23
13	Limonene	1032	1031	0.82	-
14	Eucalyptol	1035	1035	0.63	-
15	Gamma-terpinene	1063	1062	4.42	6.04
16	Sabinene hydrate	1071	1067	0.51	-
17	Terpinolene	1090	1089	0.39	-
18	Linalool	1103	1101	11.88	17.11
19	Endo-borneol	1171	1171	0.89	-
20	Terpinen-4-ol	1181	1178	1.41	-
21	Alpha-terpineol	1200	1189	0.89	-
22	D-carvone	1263	1243	0.19	-
23	Thymol	1294	1297	5.74	-
24	Carvacrol	1315	1317	53.96	58.67
25	Caryophyllene	1428	1418	1.94	-
26	Aromandendrene	1448	1439	0.56	-
27	Humulene	1462	1455	0.11	-
28	Alloaromadendrene	1502	1478	0.41	-
29	Delta-cadinene	1530	1524	0.18	-
30	Spathulenol	1586	1578	0.2	-
31	Caryophyllene oxide	1593	1583	0.24	2.73

**Table 2:** Phytochemical composition (%) of hydrolate (H) of *Thymus vulgaris* (Tv) used in this study, via GC-MS. RI exp: retention index calculated with our experimental results; RI ref: retention index found in the literature for the same compound (source: Pherobase).

No. Peak	Compound Name	RI Exp.	RI Ref.	H (Tv)
1	1-octen-3-ol	951	979	1.71
2	Dimethylstyrene	1061	1096	0.08
3	Delta-3-carene	1071	1011	0.82
4	Camphor	1144	1143	1.54
5	Borneol	1156	1165	2.65
6	Gamma-terpinene	1168	1062	0.85
7	Allyltoluene	1176	1151	0.18
8	*p*-Menth-1-en-8-ol	1184	1189	0.49
9	Thymoquinone	1255	1252	0.09
10	Thymol	1287	1297	84.01
11	Carvacrol	1290	1317	7.55
12	Alpha-cedrene	1410	1399	0.02

**Table 3:** Phytochemical composition (%) of essential oil (EO) and hydrolate (H) of *Citrus limon* (Cl) used in this study, via GC-MS. RI exp: retention index calculated with our experimental results; RI ref: Retention index found in the literature for the same compound (source: Pherobase).

No. Peak	Compound Name	RI Exp.	RI Ref.	EO (Cl)	H (Cl)
1	Beta-thujene	930	931	0.77	-
2	Alpha-pinene	937	939	3.31	3.12
3	Camphene	952	953	0.11	-
4	Beta-pinene	981	975	18.09	20.60
5	Beta-myrcene	992	991	2.35	2.72
6	Alpha-phellandrene	1005	1005	0.15	-
7	4-carene	1019	1013	0.34	-
8	Cymene	1028	1026	0.74	-
9	Limonene	1039	1031	53.37	53.45
10	Beta-ocimene	1052	1050	0.19	-
11	Gamma-terpinene	1065	1062	12.55	14.03
12	Terpinolene	1090	1089	0.67	0.70
13	Linalool	1100	1101	0.26	0.30
14	Nonanal	1105	1102	0.11	-
15	Citronellal	1156	1158	0.13	-
16	Terpinen-4-ol	1181	1178	0.09	0.10
17	Alpha-terpineol	1193	1189	0.30	0.35
18	Neral	1245	1242	1.15	1.28
19	Citral	1274	1271	1.95	2.24
20	(−) -Lavandulyl acetate	1366	1288	0.85	-
21	(+) -Lavandulyl acetate	1385	1298	0.49	-
22	Caryophyllene	1427	1418	0.33	-
23	Alpha-bergamotene	1441	1486	0.66	-
24	Valencene	1499	1495	0.10	-
25	Beta-bisabolene	1512	1509	0.94	1.02

**Table 4:** In vitro effect of *C. limon, T. vulgaris, T. serpyllum* hydrolates (H), and of *C. limon, T. serpyllum* EOs on MNV-1 infectivity immediately after treatment and after 24 h of incubation at 20 ± 2 °C by calculating log reduction value (LRV).

Treatment	Viral Titre at t = 0 (log TCDI_50_/mL ± SD)	LRV Immediately after Treatment (t = 0) (log TCDI_50_/mL ± SD)	Viral Titre at t = 24 h (log TCDI_50_/mL ± SD)	LRV after 24 h of Treatment (log TCDI_50_/mL ± SD)
Untreated MNV-1	5.4 ± 0.1	-	4.2 ± 0.3	
H-*C. limon* 1%	4.5 ± 0.2	0.9 ± 0.3	3.3 ± 0.3	0.9 ± 0.6
H-*C. limon* 2%	4.3 ± 0.2	1.1 ± 0.3	3.1 ± 0.2	1.1 ± 0.5
H-*T. vulgaris* 1%	3.5 ± 0.1	1.9 ± 0.2	3.5 ± 0.2	0.7 ± 0.5
H-*T. vulgaris* 2%	3.4 ± 0.1	2.0 ± 0.2	3.4 ± 0.1	0.8 ± 0.4
H-*T. serpyllum* 1%	3.4 ± 0.1	2.0 ± 0.2	3.3 ± 0.2	0.9 ± 0.5
H-*T. serpyllum* 2%	3.6 ± 0.1	1.8 ± 0.2	3.2 ± 0.2	1.0 ± 0.5
EO-*C. limon* 0.5%	5.3 ± 0.3	0.1 ± 0.4	4.4 ± 0.1	0
EO-*C. limon* 1%	4.1 ± 0.2	1.3 ± 0.3	3.4 ± 0.1	0.8 ± 0.4
EO-*T. serpyllum* 0.5%	5.2 ± 0.2	0.2 ± 0.3	4.3 ± 0.3	0
EO-*T. serpyllum* 1%	3.5 ± 0.1	1.9 ± 0.2	3.5 ± 0.2	0.7 ± 0.5

## Data Availability

Not applicable.
